# HyperGCN: an effective deep representation learning framework for the integrative analysis of spatial transcriptomics data

**DOI:** 10.1186/s12864-024-10469-x

**Published:** 2024-06-05

**Authors:** Yuanyuan Ma, Lifang Liu, Yongbiao Zhao, Bo Hang, Yanduo Zhang

**Affiliations:** 1 School of Computer Engineering, Hubei University of Arts and Science, Xiangyang, China; 2 Hubei Key Laboratory of Power System Design and Test for Electrical Vehicle, Hubei University of Arts and Science, Xiangyang, China; 3 School of Physics and Electronic Engineering, Hubei University of Arts and Science, Xiangyang, China; 4 School of Computer, Central China Normal University, Wuhan, China

**Keywords:** Hypergraph convolutional network, Spatial transcriptomics, Single cell multi-omics, Integrative analysis

## Abstract

**Background:**

Advances of spatial transcriptomics technologies enabled simultaneously profiling gene expression and spatial locations of cells from the same tissue. Computational tools and approaches for integration of transcriptomics data and spatial context information are urgently needed to comprehensively explore the underlying structure patterns. In this manuscript, we propose HyperGCN for the integrative analysis of gene expression and spatial information profiled from the same tissue. HyperGCN enables data visualization and clustering, and facilitates downstream analysis, including domain segmentation, the characterization of marker genes for the specific domain structure and GO enrichment analysis.

**Results:**

Extensive experiments are implemented on four real datasets from different tissues (including human dorsolateral prefrontal cortex, human positive breast tumors, mouse brain, mouse olfactory bulb tissue and Zabrafish melanoma) and technologies (including 10X visium, osmFISH, seqFISH+, 10X Xenium and Stereo-seq) with different spatial resolutions. The results show that HyperGCN achieves superior clustering performance and produces good domain segmentation effects while identifies biologically meaningful spatial expression patterns. This study provides a flexible framework to analyze spatial transcriptomics data with high geometric complexity.

**Conclusions:**

HyperGCN is an unsupervised method based on *hyper*graph induced *g*raph *c*onvolutional *n*etwork, where it assumes that there existed disjoint tissues with high geometric complexity, and models the semantic relationship of cells through hypergraph, which better tackles the high-order interactions of cells and levels of noise in spatial transcriptomics data.

**Supplementary Information:**

The online version contains supplementary material available at 10.1186/s12864-024-10469-x.

## Background

The development of spatial transcriptomics technologies enables genome-wide profiling of transcriptional expressions in captured relative locations at a resolution of several cells or even individual cell level, such as 10X Visium [1], 10X Xenium, Slide-seq [2, 3], Stereo-seq [4], osmFISH [5], PIXEL-seq [6], SeqFISH+ [7], and Seq-Scope [8]. Compared with nonspatial single-cell RNA-sequencing technologies, spatial transcriptomics can capture cellular heterogeneity coupled with its spatial coordinates in the same tissue, which provide the rich biological insights of cell functions and their cross-talk [9, 10]. Integrating gene expression and spatial coordinate information to learn a good representation for spatial transcriptomic data analysis is crucial. Computational tools and approaches are urgently needed to dissect spatial organization domains and functions of individual cells.

Increasing evidences have shown that some cell types, such as neurons and endothelia cells have high heterogeneities and specific spatial expression patterns [5, 11, 12]. Even for cells with the same type, such as ependymal cells, high spatial self-affinity was also observed. In addition, spatial self-evasion was measured in microglia and astrocytes inhibitory neurons [5]. Therefore, spatial neighbors of each cell may provide valuable information for understanding cell heterogeneity and annotating tissue domains. However, some single-cell integration methods which are initially designed for nonspatial single-cell multi-omics data [13–16] cannot employ spatial information to enhance their analytical ability. This situation poses significant challenges in spatial data analysis.

Recently, several new computational approaches have been developed for spatial transcriptomics data analysis [9, 17–24]. SpaGCN integrates gene expression, spatial coordinates, and histological information into an undirected weighted graph, and then employs graph convolution to cluster theses spots into different spatial domains [18]. stLearn utilizes a deep neural network (CNN) on the morphological data to extract low-dimensional morphological features, on which the morphological similarities between neighboring spots are computed [19]. Then, the normalization of gene expression matrix is established based on the morphological similarities and spatial neighbors of each spot, followed by dimensionality reduction with PCA and UMAP [25]. BayesSpace assumes that the spots containing the same cell type should be closer to each other in space, and trains the models with Bayesian statistical approach [21]. SpaceFlow uses the deep graph infomax (DGI) framework where contrastive learning strategy is used to train the graph encoder. Simultaneously, a spatial regularization term is added into the objective function of DGI to preserve the spatial consistency of the low-dimensional spot embeddings [20]. SEDR uses a deep autoencoder network and a variational graph autoencoder network to learn the low-dimension representation of transcriptomic profile matrix, where spatial information is used to construct neighborhood graph [9]. Notably, these approaches that employ GCNs (including SpaGCN, SEDR and SpaceFlow) mainly rely on similarity graph calculated based on the *k-*nearest neighbors (*k*NN) of each spot, and ignore high-order structure information in disjoint tissues with high geometric complexity, which limits their application to unknown and complicated data. Compared with SpaGCN and SpaceFlow, one drawback of stLearn is that linear PCA is used to conduct dimension reduction for the normalized gene expression matrix, and it cannot model complex non-linear relationships among cells. The disadvantage of BayesSpace is the interpretability: it does not generate jointly embeddings of gene expression and spatial coordinates, hindering its application into some downstream analysis tasks.

**Fig. 1:**
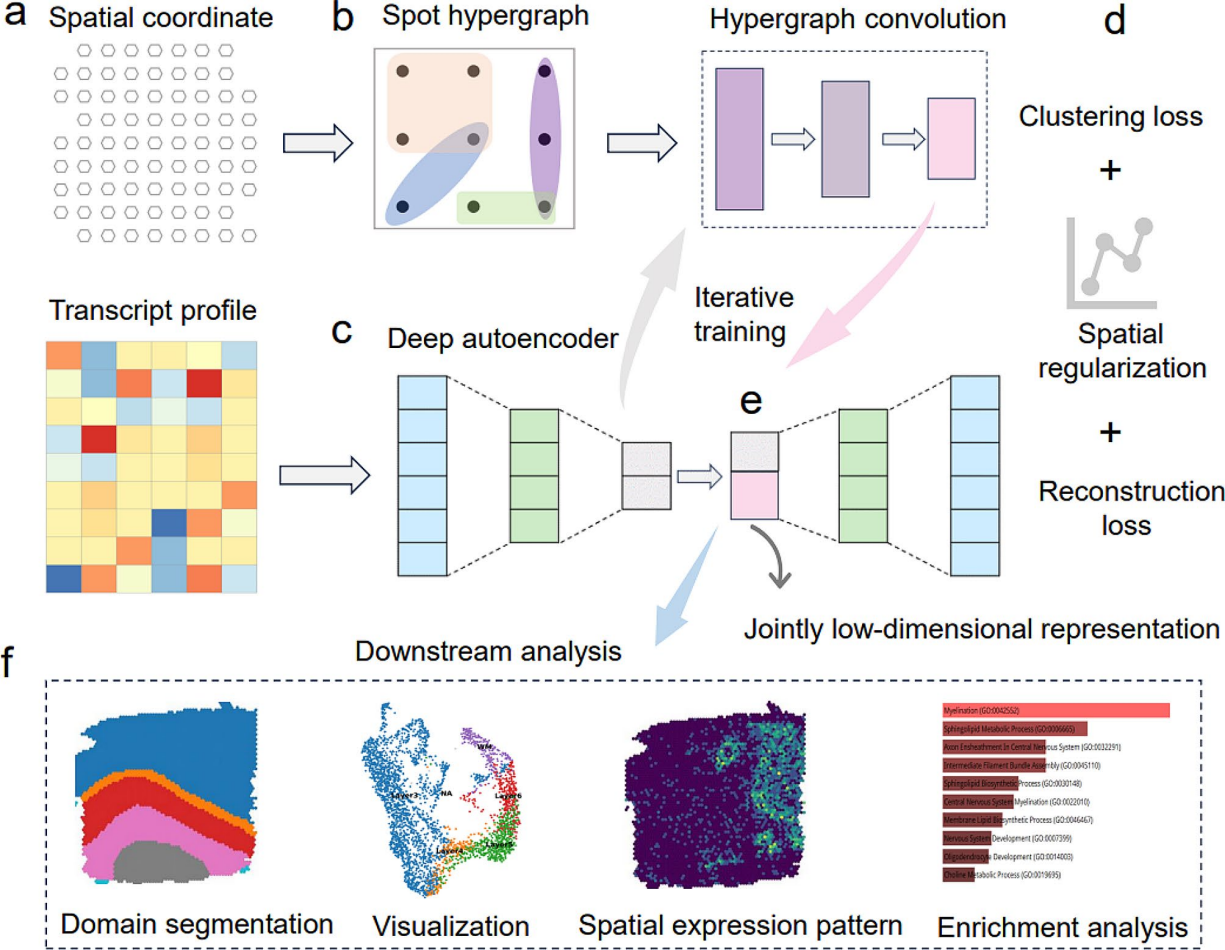
Overview of HyperGCN. (**a**) The inputs of HyperGCN are a gene expression matrix and spatial coordinates of spots/cells. (**b**) A hypergraph is constructed based on the spatial information and is used as input of hypergraph convolutional network. (**c**) A deep autoencoder network encodes the gene expression into a low-dimensional representation space. Simultaneously, a two-layer variational hypergraph convolutional network is utilized to generate a spatial embedding. The generated spatial embedding is then concatenated with the low-dimensional representation to form the jointly latent embedding that is used to reconstruct the original gene expression. (**d**) The joint embedding is regularized to preserve spatial consistency. Clustering is implemented on the joint embedding. Reconstruction loss for gene expression matrix, deep embedding clustering (DEC) loss and regularization loss are optimized simultaneously until convergence. (**e**) The joint embedding obtained from the trained encoder. (**f**) The output of HyperGCN can be applied for domain segmentation, UMAP visualization, exploring spatial gene expression pattern and GO enrichment analysis

In this work, we proposed HyperGCN for the integrative analysis of spatial transcriptomics data, where both gene expression and spatial locations of spots are simultaneous measured. HyperGCN is a versatile tool that enables accurate clustering of spots/cells and data visualization, and it facilitates the downstream analysis, including the identification of layer structures, the characterization of domain-specific marker genes, and biological processes and functional pathways enrichment analysis. HyperGCN is a novel computational framework, referring to *Hyper*graph induced *G*raph *C*onvolutional *N*etwork. Unlike SpaGCN and SEDR which utilize nearest neighbor information to encode spatial proximity between spots, HyperGCN assumes that there existed disjoint tissues with high geometric complexity, and models the semantic relationship of cells through hypergraph convolution and spatial regularization, which better tackles the high-order interaction of cells and levels of noise in spatial transcriptomics data. HyperGCN not only integrates the complementary information from transcriptomic data and spatial coordinates, but also preserves the geometric structures in original high dimensional space. We applied HyperGCN to four real spatial transcriptomics datasets from different tissues tissues (including human dorsolateral prefrontal cortex, human positive breast tumors, mouse brain, mouse olfactory bulb tissue and Zabrafish melanoma) and technologies (including 10X visium, osmFISH, seqFISH+, 10X Xenium and Stereo-seq), the results show that HyperGCN is effective in spatial transcriptomics data analysis: HyperGCN achieves superior performance in clustering and domain segmentation, and it captures and enhances domain structures that were not easily identified by other methods. The clustering assignments obtained from HyperGCN have latent biological application and meaning: the top domain-specific genes for each layer show spatial specific expression patterns with high expression level in this domain and low expression outside this domain. Moreover, it also provided rich information on the biological interpretation of the markers. Gene Ontology (GO) enrichment analysis indicates that enriched biological processes (BPs) are directly related to the biological functions of the underlying structured domains. An overview of HyperGCN is shown in Fig. 1a-f.

## Materials and methods

### Datasets and data preprocessing

Four real spatial transcriptomics data were analyzed in this study and can be downloaded from their original publications. Specifically, (1) The LIBD human dorsolateral prefrontal cortex (DLPFC) data was sequenced by the 10x Genomics Visium technique [26]. It contains 12 spatially resolved RNA-seq data sets, and it was downloaded from the website (http://research.libd.org/spatialLIBD/); (2) The osmFISH dataset of the somatosensory cortex sequenced with osmFISH technique [5] was downloaded from the website (http://linnarssonlab.org/osmFISH/); (3) The 10X Visium mouse brain sagittal data was downloaded from 10X genomics website (https://www.10xgenomics.com/spatial-gene-expression/datasets). (4) The Stereo-seq data from mouse olfactory bulb tissue was downloaded from SEDR website (https://github.com/JinmiaoChenLab/SEDR_analyses).

The raw gene expression data were preprocessed with the SCANPY package [27]. Firstly, the genes that expressed in less than 5 spots/cells are filtered out. Secondly, the counts are normalized such that the total counts of all genes in each spot/cell equal to 1. To alleviate the effect of extreme values, the entries in the matrix were log-transformed with a pseudo-count of 1, and scaled to have unit variances and zero means. Finally, we used PCA with 200 principal components to implement dimension reduction on the normalized expression data. For osmFISH data with only 33 genes, we do not conduct PCA as the low dimension of features.

The detailed statistics of these datasets is presented in Additional file 1: Supplementary Table S1.

### Hypergraph construction for spatial transcriptomics data

The previous studies assumed that there existed pairwise relationships among the spots/cells [9]. A simple graph is generally used to describe the pairwise relationships. In this graph, two spots/cells are connected by an edge if they are adjacent in space. However, in many real problems, it may cause information loss to represent a group of complex objects only by using simple graph [28]. For example, to group members within one club into different communities, we first construct a simple graph where two members are connected if they share the same coach. Then, clustering methods based on spectral graph are applied [29, 30]. However, this approach mentioned above may lose some useful information in the scenario where the same coach jointly teaches more than two members. Such unexpected information loss may result in the performance degradation of downstream clustering algorithms. Because these members taught by the same coach likely belong to the same community.

**Fig. 2:**
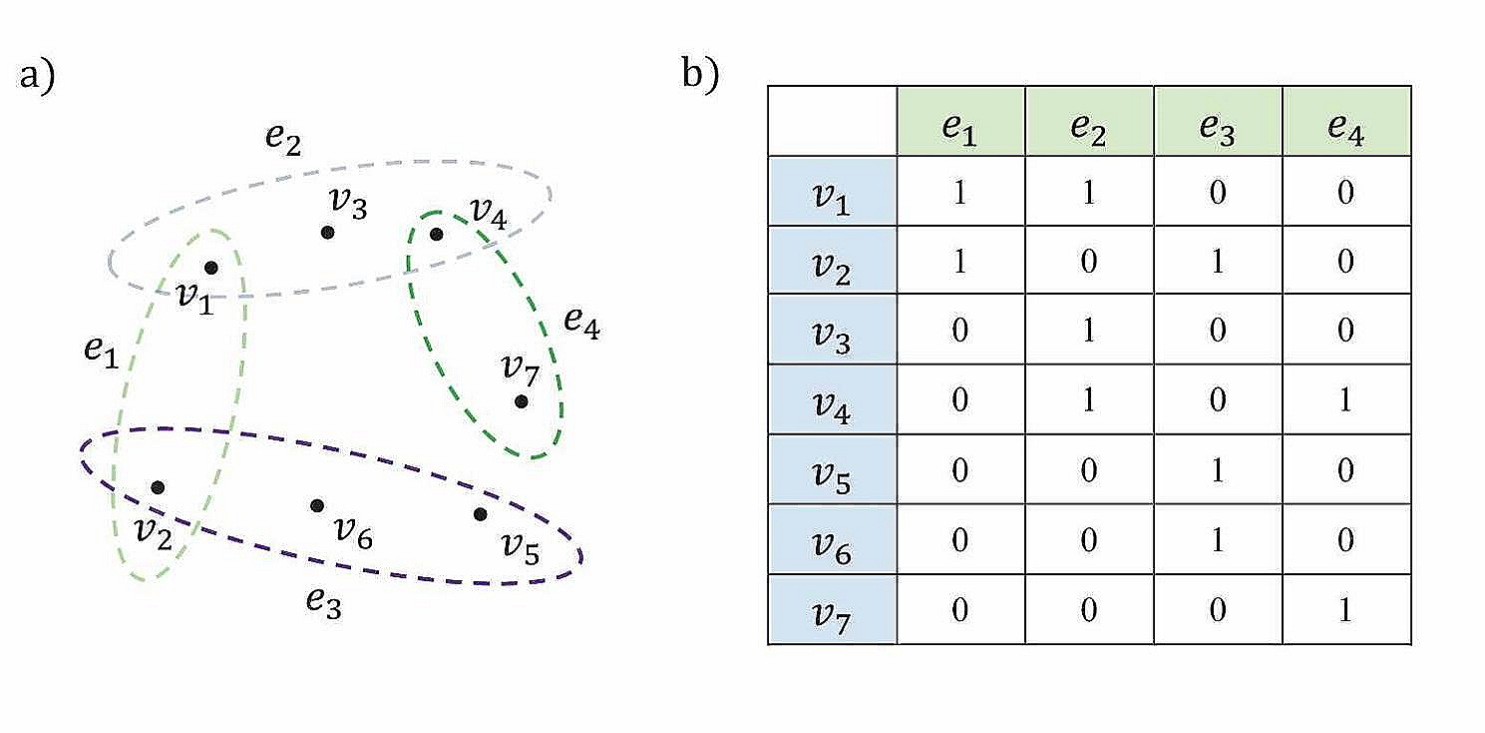
An illustrative example of hypergraph. (**a**) In hypergraph, a hyperedge *e* can connect more than two nodes. The hypergraph is constructed based on these hyperedges. (**b**) The incidence matrix of hypergraph, where the entry (${v}_{i}$, ${e}_{j}$) equals to be 1 when ${v}_{i}$ belongs to ${e}_{j}$, and 0 otherwise

A natural way to deal with the information loss issue mentioned above is to represent the high-order relationships by using hypergraph (Fig. 2a-b).

Let $V$ denotes the set of spots/cells and $E$ be a family of subset of $V$. For any hyperedge $e$, ${\bigcup }_{e\in E}=V$. The weight corresponding to each hyperedge $e$ are denotes as $w\left(e\right)$. A weighted hypergraph is represented as $G=\left(V,E,W\right)$. The incidence matrix $P\in {R}^{\left|V\right|\times \left|E\right|}$ corresponding to $G$ is defined as the following.

1 $$p\left(v,e\right)=\left\{\begin{array}{c}1, if v\in e,\\ 0, if v\notin e.\end{array}\right.$$

$\left|V\right|$ indicates the number of vertices. $\left|E\right|$ is the number of hyperedges. Given spot $v\in V$ and hyperedge $e\in E$, their degrees are defined as $d\left(v\right)={\sum }_{e\in E}w\left(e\right)p\left(v,e\right)$ and $\delta \left(e\right)=\left|e\right|$, respectively. Let ${D}_{v}$ and ${D}_{e}$ denote degree matrices for spots and hyperedges, ${W}_{e}$ denote the weight matrix of hyperedges. Then, the normalized hypergraph Laplacian matrix can be formulated as:

2 $${L}_{hp}={{I-D}_{v}}^{-\frac{1}{2}}P{W}_{e}{{D}_{e}}^{-1}{P}^{T}{{D}_{v}}^{-\frac{1}{2}}$$

The normalized adjacency matrix of hypergraph can be formulated as the follows, and is used to the inputs of HyperGCN together with spatial transcriptomics data.

3 $${A}_{hp}={{D}_{v}}^{-\frac{1}{2}}P{W}_{e}{{D}_{e}}^{-1}{P}^{T}{{D}_{v}}^{-\frac{1}{2}}.$$

where, ${P}^{T}$ denotes the transposition of incidence matrix $P$. In contrast to the previous study [31], we add self-similarity to each node to maintain the numerical stability.

Note that we first use the spatial coordinates of spots to construct the *k*NN graph, and then a hypergraph is constructed based on the *k*NN graph with the above methodology. In the whole experiments, we set $k=20$ for all the datasets and ${W}_{e}$ as identity matrix.

The sensitivity analysis of the hyperparameter $k$ are presented in Additional file 1.

### Deep autoencoder for low-dimensional representation learning

The low-dimensional representation of spots/cells is learned by using a deep autoencoder. In the encoder part, two fully connected linear layers with Elu activation function are stacked together, and produces a low-dimensional spot/cell embedding matrix ${H}_{f}\in {\mathbb{R}}^{N\times {D}_{f}}$ from the preprocessed transcript profile matrix $X\in {\mathbb{R}}^{N\times M}$. In the decoder part, one fully connected linear layer is used to reconstruct the transcript matrix${X}^{{\prime }}\in {\mathbb{R}}^{N\times M}$ from the latent spot/cell representation matrix$H\in {\mathbb{R}}^{N\times D}$ which is generated by concatenating the low-dimensional spot/cell embedding ${H}_{f}$ and spatial embedding ${H}_{g}\in {\mathbb{R}}^{N\times {D}_{g}}$ (obtained from hypergraph convolutional network). Here, $N$ is the number of spots/cells, $M$ is the number of features, and ${D}_{f}$, ${D}_{g}$ are dimensions of features for autoencoder and spatial embedding, respectively.$D={D}_{f}+{D}_{g}$ is the final feature dimension learned from HyperGCN.$H\in {\mathbb{R}}^{N\times D}$ is applied into various downstream analysis tasks.

The deep autoencoder aims to minimize the loss between the input transcript profile matrix $X$ and the reconstructed matrix ${X}^{{\prime }}$. The objection function is defined as the following.

4 $${Loss}_{rec}=MSE(X,{ X}^{{\prime }})$$

where $MSE\left(*\right)$ denotes the mean squared error loss function.

### Hypergraph convolution for high-order spatial embedding of spots/cells

In contrast to simple graph, hypergraph encodes the high-order spatial relationships among spots/cells, and is able to identify the latent spatial domain. In terms of the good performance of graph convolutional network [18, 32], we use hypergraph convolution to embed the spatial information of neighboring spots/cells.

Given the normalized adjacency matrix of hypergraph ${A}_{hp}$ and corresponding weight matrix ${W}_{i}$, the two-layer hypergraph convolutional networks is defined as the following.

5 $$HGCN({A}_{hp}, {H}_{f})= {A}_{hp}ReLU\left({A}_{hp}{H}_{f}{W}_{1}\right){W}_{2}$$

where${H}_{f}$ is a low-dimensional representation of spots/cells obtained from the deep autoencoder. To enhance the representation ability of ${H}_{g}$, we introduce the variational graph autoencoder(VGAE) [33] framework. The VGAE utilizes latent variables and learns an interpretable and meaningful embedding with the following function: $g:\left({A}_{hp}, {H}_{f}\right)\to {H}_{g}$. The inference model of VGAE parameterized by (5) is defined as:

6 $$g\left({H}_{g}|{A}_{hp},{H}_{f}\right)={\prod }_{i=1}^{N}g\left({h}_{i}\right|{A}_{hp},{H}_{f})$$

7 $$g\left({h}_{i}|{A}_{hp},{H}_{f}\right)=\mathcal{N}\left({h}_{i}\right|{\mu }_{i},diag\left({\sigma }_{i}^{2}\right))$$

Here, $\mu ={HGCN}_{\mu }({A}_{hp},{H}_{f})$ is the matrix of mean vectors ${h}_{i}$, and $log\sigma ={HGCN}_{\sigma }({A}_{hp},{H}_{f})$.

In the proposed HyperGCN model, we only train the autoencoder with reconstruction loss of the input gene expression matrix $X$, and do not consider the VGAE loss.

### Hypergraph induced deep autoencoder clustering framework

HyperGCN implements an unsupervised deep embedded clustering on the low-dimensional embedding $H$ of the autoencoder [34]. To enhance the initialization step of clustering, *k-*means is employed to generate the centroids. The number of centroids in HyperGCN is set as 10 for all datasets.

i. Deep embedding clustering.

Deep embedding clustering (DEC) employs the Kullback–Leibler (KL) divergence to measure the clustering consistence between the soft assignment $Q$ and the auxiliary target distribution $T$. The objective function is defined as the following.

8 $${Loss}_{clu}=KL\left(T?Q\right)=\sum _{i}\sum _{k}{t}_{ik}log\frac{{t}_{ik}}{{q}_{ik}},$$

where the soft assignment ${q}_{ik}$ indicates the distance between spot/cell ${h}_{i}$ and cluster center ${\mu }_{k}$, and is calculated by Student’s *t*-distribution [35]:

$${q}_{ik}=\frac{{\left(1+{?{h}_{i}-{\mu }_{k}?}^{2}\right)}^{-1}}{{\sum }_{{k}^{{\prime }}}{\left(1+{?{h}_{i}-{\mu }_{k{k}^{{\prime }}}?}^{2}\right)}^{-1}}.\left(9\right)$$

The auxiliary target distribution $T$ refines the clusters by emphasizing the higher confidence assignments. Formally, ${t}_{ik}$ is defined as the following.

$${t}_{ik}=\frac{\frac{{q}_{ik}^{2}}{{\sum }_{i}{q}_{ik}}}{{\sum }_{{k}^{{\prime }}}\left(\frac{{q}_{i{k}^{{\prime }}}^{2}}{{\sum }_{i}{q}_{i{k}^{{\prime }}}}\right)}.\left(10\right)$$

HyperGCN iteratively refines the clusters with the clustering loss (8) and improves the initial estimate of centroids from *k*-means. Thus, a high confident spot/cell assignments are learned.

ii. Enhancing spatial consistency with spatially regularization.

The closeness in the embedding space not only reflects the transcript similarity between spots/cells, but also their spatial proximity [20]. To enhance the spatial consistency among spots/cell, a spatial regularization term is defined as follows.

$${Loss}_{spa}={\sum }_{i=1}^{N}{\sum }_{j=1}^{N}\frac{{D}_{ij}^{\left(s\right)}*\left(1-{D}_{ij}^{\left(h\right)}\right)}{{N}^{2}},\left(11\right)$$

where${D}_{ij}^{\left(s\right)}$ is the Euclidean spatial distance between spot/cell $i$ and $j$,${D}_{ij}^{\left(h\right)}$ denotes the embedding distance between $i$ and $j$. Obviously, the spots or cells that are spatially distant, are also pushed further from each other in the generated embedding space by imposing the spatial regularization term (11). Strong spatial regularization makes the embeddings more smooth, which may not accord with more complicated biological heterogeneity. To address the problem mentioned above, regularization parameter $\gamma$ is introduced into Eq. 11 to control the strength of spatial regularization. Over-smoothing or undersmoothing issues may have an significant influence on the performance of domain segmentation and clustering, so tuning of the parameter $\gamma$ is rigamarole. In the whole experiments, we experientially set $\gamma =1$ to simplify analysis.

Combining the reconstruction loss, clustering loss and spatial regularization, the final object of HyperGCN is defined as follows.

12 $$L={{Loss}_{rec}+\lambda Loss}_{clu}+{\gamma Loss}_{spa},$$

where $\lambda$, $\gamma$ are parameters for the clustering loss and spatial regularization term, respectively. In the whole experiments, we set $\lambda =0.1$, $\gamma =1$ across all datasets.

### Training procedure

We use the Adam optimizer with a default learning rate $lr=0.01$ to train HyperGCN. The number of epochs is set as 200. The *k*NN graph is obtained via “kneighbors_graph” function from scikit-learn package. We use Elu activation function in the autoencoder, in view of its advantages compared other activation functions (Additional file 1). We pretrain the autoencoders without DEC loss for 200 epochs. In the beginning of the DEC stage, we utilize *k*-means to enhance the initialization step of cluster centroids. The number of centroids in *k*-means is empirically set as 10 for all datasets. For hypergraph convolution layers, the dimensions of hidden layers are set to be 32 and 8, respectively. For autoencoder layers, the dimensions of hidden layers are set to be 100 and 20, respectively. During the clustering stage, the clustering loss, reconstruction loss are optimized together with spatial regularization loss. A GeForce RTX 3060 Laptop GPU with 6G memory is used for training the HyperGCN model.

### Competing methods

We compare HyperGCN with several recently published methods on spatial transcriptomics data, including SpaGCN [18], BayesSpace [21], SEDR [9] and SpaceFlow [20]. In the experiments, the numbers of clusters are set as the numbers of annotated layers for DLFPC data and osmFISH data. For 10X Visium data and Stereo-seq data, we set the numbers of cluster equal to 12 and 7, respectively.

When benchmarking with SpaGCN, the recommended parameter setting described in their online publishment, such as $s=1$, $b=49$ and$p=0.5$ are used across all datasets.

For BayesSpace benchmarking, the getTopHVGs method is used to select the highly variable genes (HVGs, top 2000 exclude osmFISH data), the spatialPreprocess method is used to log-normalize the count matrix and runPCA method is used for dimension reduction. Then, the spatialCluster function is used to cluster spots/cells with 5000 MCMC iterations, and defaulted gamma for different sequencing platforms.

For SEDR benchmarking, we used the default parameters described in their online tutorial (https://github.com/JinmiaoChenLab/SEDR/), including $epochs=200$, $lr=0.01$, $k=50$.

For SpaceFlow benchmarking, we used the default parameters setting and data preprocessing method provided in their online tutorial (https://github.com/hongleir/SpaceFlow).

### Evaluation metrics

The clustering performances of different models are evaluated with Adjusted Rand Index(ARI) [36], Normalized Mutual Information (NMI) [37] and Moran’s Index [38].

Let $G$ denote the ground truth spot/cell labels, and $P$ denote the predicted clustering assignments. ARI is computed as:

13 $$ARI\left( {G,P} \right) = {{{{\sum\limits_{ij} {\left( {\matrix{{{N_{ij}}} \cr 2 \cr} } \right)} - \left[ {\sum\limits_i {\left( {\matrix{{{N_i}} \cr 2 \cr} } \right)} \sum\limits_j {\left( {\matrix{{{N_j}} \cr 2 \cr} } \right)} } \right]} \over {\left( {\matrix{ N \cr 2 \cr} } \right)}}} \over {{{{1 \over 2}\left[ {\sum\limits_i {\left( {\matrix{{{N_i}} \cr 2 \cr} } \right)} + \sum\limits_j {\left( {\matrix{{{N_j}} \cr 2 \cr} } \right)} } \right] - \left[ {\sum\limits_i {\left( {\matrix{{{N_i}} \cr 2 \cr} } \right)} \sum\limits_j {\left( {\matrix{{{N_j}} \cr 2 \cr} } \right)} } \right]} \over {\left( {\matrix{ N \cr 2 \cr} } \right)}}}}$$

where $N$ is the number of spots/cells and ${N}_{ij}$ is the number of spots/cells of class label ${C}_{j}^{*}\in G$ assigned to cluster ${C}_{i}$ in partition $P$. ${N}_{i}$ is the number of spots/cells in cluster ${C}_{i}$ of partition $P$, and ${N}_{j}$ is the number of spots/cells in class ${C}^{*}$. NMI is computed as the following:

$$NMI\left(G,P\right)=\frac{MI\left(G,P\right)}{\sqrt{H\left(G\right)H\left(P\right)}},\left(14\right)$$

where $MI\left(G,P\right)$denotes the mutual information between $G$ and $P$, $H\left(G\right)$ and $H\left(P\right)$denote the information entropy of $G$ and $P$, respectively.

ARI and NMI measure the consistency between two sets. Generally, high ARI and NMI values indicate good performance. Acknowledge that the spot/cell annotated in the original publications may not be fully accurate, we also used a variant of Moran’s Index to evaluate the clustering performance [39]. Moran’s Index does not require true labels and is defined as:

$${I}^{label}=\frac{N}{{\sum }_{i=1}^{N}{\sum }_{j=1}^{N}A}\frac{{\sum }_{i=1}^{N}{\sum }_{j=1}^{N}{A}_{ij}{B}_{ij}}{N},\left(15\right)$$

$${B}_{ij}=\left\{\begin{array}{c}1, if {y}_{i}={y}_{j} \\ 0, otherwise\end{array},\right.\left(16\right)$$

where $N$ is the number of spots/cells, $A$ is the *k*NN graph ($k=20$) calculated using spatial coordinate information of spots.${I}^{label}$ measures the cell-type spatial concentration. In other words, spots that are close in physical space should also be assigned the same label. A high ${I}^{label}$ score indicates good performance. The values of ARI, NMI and ${I}^{label}$ range from 0 to 1.

## Results and discussion

### HyperGCN leads to improved clustering performance on four real spatial transcriptomics data from different tissues and technologies

We first assessed the clustering performance of HyperGCN on four spatial transcriptomics datasets. These datasets include human DLPFC data and mouse brain sagittal data that are sequenced by 10X Visium technique; the somatosensory cortex data with osmFISH technique and the Stereo-seq data from mouse olfactory bulb tissue.

We compared HyperGCN with four existing methods for spatial transcriptomic data analysis, including SpaGCN [18], BayesSpace [21], SEDR [9] and SpaceFlow [20]. For SpaGCN and BayesSpace, we implemented its default data preprocessing and clustering methods with the recommended parameters. For SpaceFlow and SEDR, we implement Leiden clustering [40] on the generated embeddings. For HyperGCN, we first constructed hypergraph with *k*NN ($k=20$) using the spatial information of spots/cells, and then trained the model and implemented Leiden clustering algorithm on the embeddings.

**Fig. 3:**
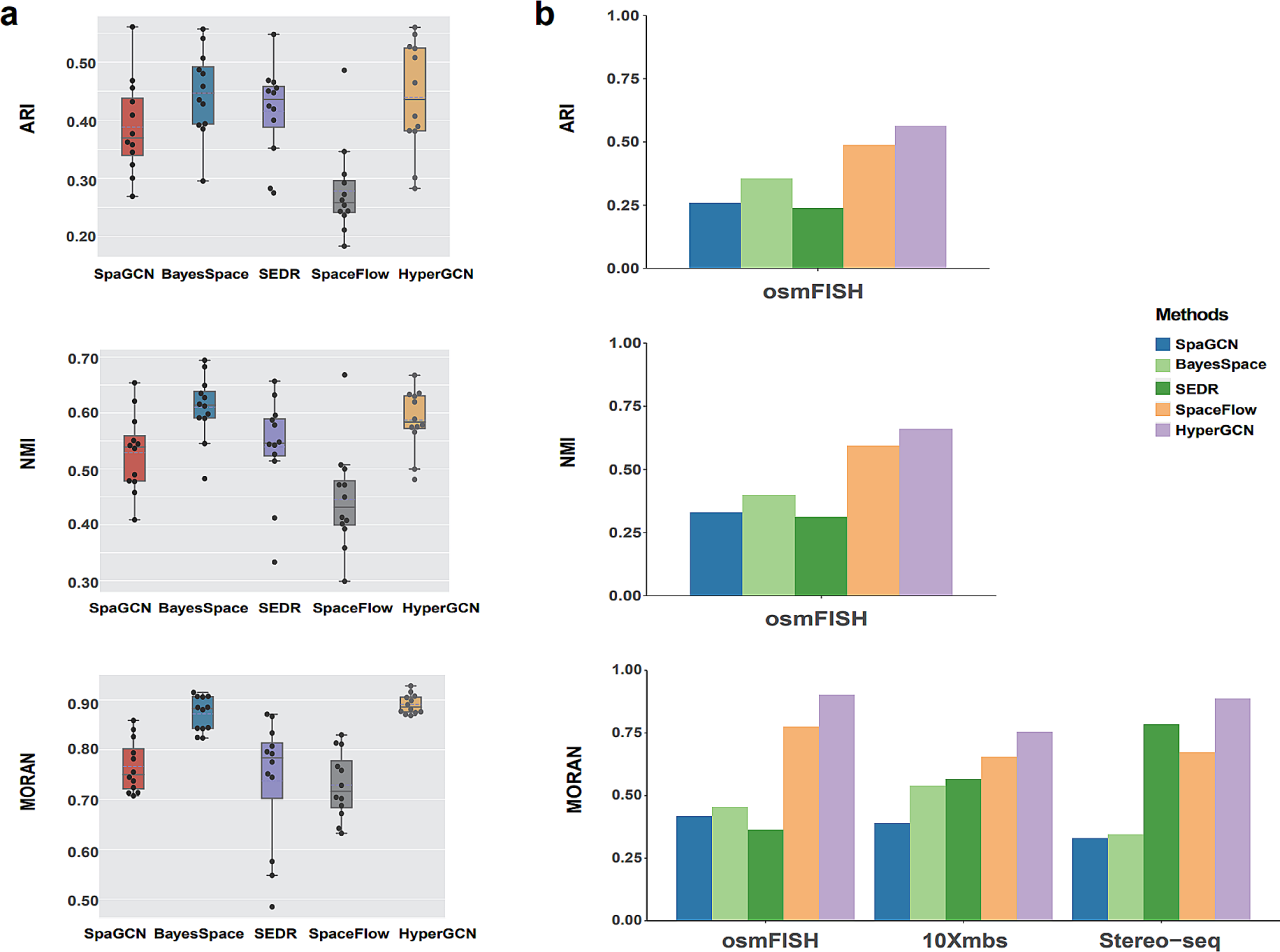
Assessment of clustering performance. (**a**) Comparison of clustering results with ARI, NMI and Moran’s Index on DLPFC data with 12 spatially resolved RNA-seq data sets. Boxplots of these metrics are presented. (**b**) Comparison of clustering results on osmFISH, 10Xmbs and Stereo-seq datasets. In computing ARI and NMI, the annotated layers of spots/cells in the original publications are used as the true labels. The Moran’s Index quantifies the spatial correlation of spots/cells, and does not require the ground truth spot/cell labels. All methods implement either the default clustering or Leiden clustering

The clustering performance evaluated by ARI, NMI and Moran’s Index is shown in Fig. 3a-b. ARI and NMI are calculated based on the annotated layers in the original publishments, and Moran’s Index is calculated based on the generated clustering assignments and does not require the true labels.

As shown in Fig. 3, we can see that the proposed HyperGCN algorithm performs well on four datasets in terms of ARI, NMI and Moran’s Index. For the DLPFC data, BayesSpace also performs well in terms of ARI and NMI. For the clustering performance evaluated by the Moran’s Index, HyperGCN is the best among all methods. For the osmFISH dataset, HyperGCN significantly outperforms other methods on three clustering metrics. For the 10Xmbs data and the Stereo-seq data, which do not have the ground truth labels, HyperGCN also performs well in Moran’s Index. The numeric values of the clustering metrics are also provided in Additional file 1: Supplementary Table S2.

To further demonstrate the effectiveness and efficiency of our proposed HyperGCN, we also implemented extensive experiments on four datasets from different sequencing techniques and tissues, including seqFISH+(mouse brain cortex) [7], ST(human positive breast tumors) [41], 10X Xenium(human colon cancer) and 10X Visium(Zabrafish melanoma). In addition, we have also benchmarked with other state-of-the-art methods, including SOTIP [24], STAGATE [23], GraphST [22] and DR_SC [17] on different datasets. The experimental results showed that HyperGCN achieved the consistently superior performance on most cases. The results are presented in Additional file 1: Supplementary Table S3 and S4.

In the construction of hypergraph, we used the *k*NN graph to generate hypergraph. In the whole experiments, we set $k=20$ for all the datasets. Sensitivity analysis of hyperparameter $k$ showed that the different values of $k$ led to the change of performance of the model. Especially, when the number of cells is small (< 5000), the performance of HyperGCN seems less stable. However, when the scale of dataset is large, the performance of HyperGCN is relatively stable with $k$ varies (Stereo-seq data, 19,527) (Additional file 1). In the experiments, for hyperparameters selection, we set $\lambda =0.1$, $\gamma =1$ for all datasets, for other values of these two parameters, HyperGCN still has stable performance(Additional file 1: Supplementary Table S5-S6). We also tested the robustness of HyperGCN on the number of centroids in *k*-means, the experimental results showed that the performance of HyperGCN was robust to the number of centroids in most cases (Additional file 1). The numbers of clusters are set as the numbers of annotated layers for DLFPC data and osmFISH data, respectively. For 10X Visium data and Stereo-seq data, we empirically set the numbers of cluster equal to 12 and 7, respectively. We also implemented experiments to validate the robustness of HyperGCN by varying the numbers of clusters, the results showed that HyperGCN is stable in most cases (Additional file 1: Supplementary Table S7). For a dataset with unknown number of clusters, we suggest that using unsupervised Moran Index to select the number of clusters by implementing grid research.

To further test the performance of HyperGCN, we also implemented two simplified variants of model (12): (1) in the first variant, we only include spatial regularization loss in the model by letting ${\uplambda }$ to be 0; (2) in the second variant, only the clustering loss is included in the model by letting ${\upgamma }$ to be 0. The experimental results show that HyperGCN outperforms its two simplified variants in most of datasets (Additional file 1: Supplementary Table S8), which indicates that introducing DEC and spatial regularization into hypergraph autoencoder clustering framework is an effective strategy in spatial transcriptomics data analysis.

### HyperGCN improves the identification of layer structures in the DLPFC tissue

**Fig. 4:**
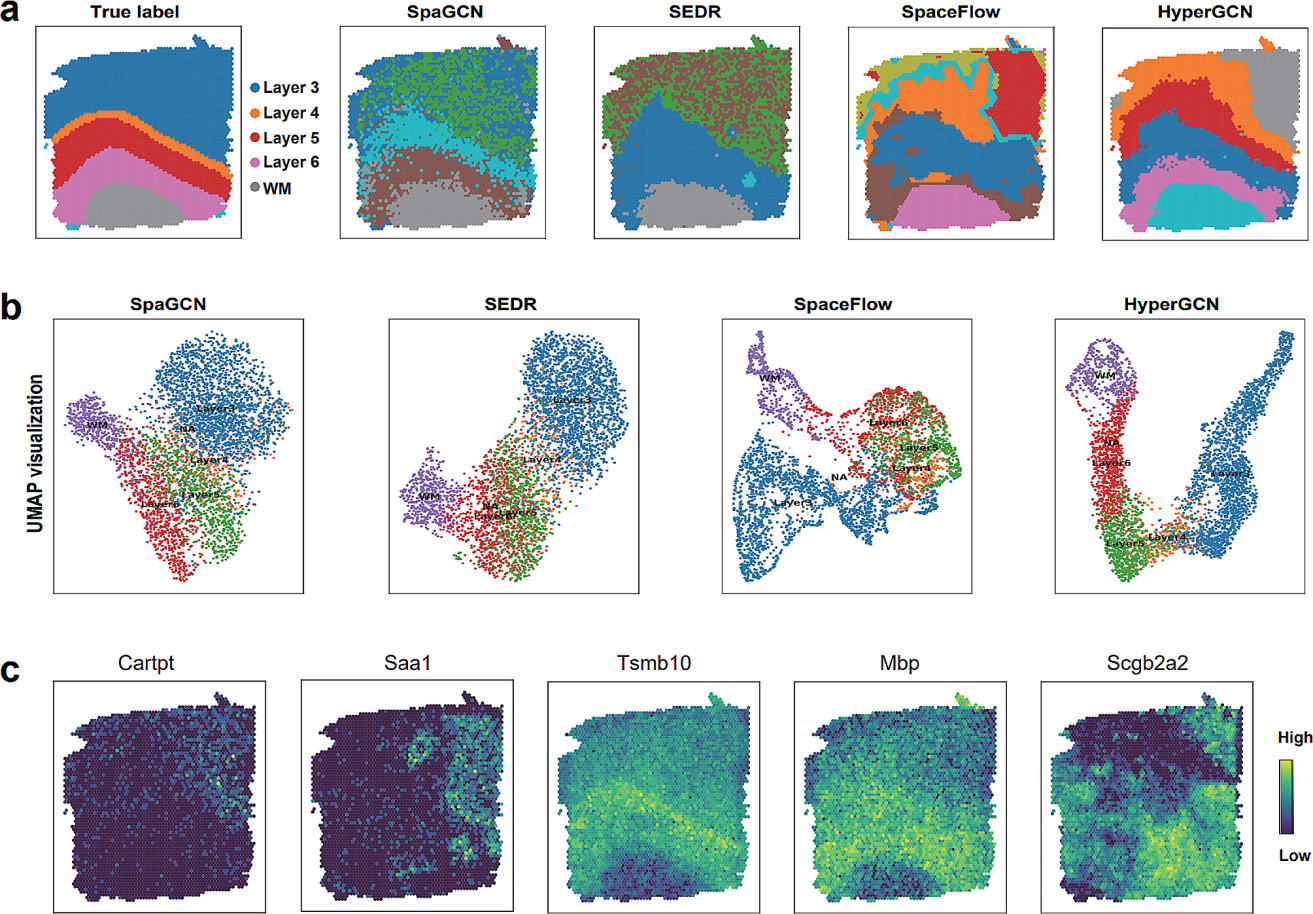
HyperGCN improves the identification of spatial domains and generates the consistent embeddings on the human dorsolateral prefrontal cortex (DLPFC) data. (**a**) Domain segmentations generated by annotated labels (top left panel) and SpaGCN, SEDR, SpaceFlow and HyperGCN using Sect. 151,671 of DLPFC data. (**b**) UMAP visualization on DLPFC data secion 151,671 by using the low-dimensional embeddings from SpaGCN, SEDR, SpaceFlow and HyperGCN. Spots are colored based on their annotated layer labels provided in the original publishment of the data. (**c**) Spatial gene expression heatmaps of HyperGCN for the top-1 markers for the identified domains

To further evaluate the clustering performance of HyperGCN embeddings, we first compute the domain segmentation for each competitive method and visualize the outputs on Sect. 151,671 of DLPFC data (Fig. 4a). The manually annotated layers and white matter (WM) are used as the ground truth [42]. It can be seen that HyperGCN captures the best layer structures. Both SpaGCN and HyperGCN can identify Layer 5, Layer 6 and WM domains observed in the annotation, but SpaGCN shows noisy boundaries between domains. SEDR identifies Layer 6 and WM domain, but is unable to capture other remaining structures (Layer 3, Layer 4, Layer 5 and Layer 6). SpaceFlow captures the WM structure, but shows irregular and non-contiguous domain structures. Interestingly, HyperGCN found a subdomain at the top right of Layer 3 (labeled in gray). This result is also consistent with the domain observed in SpaceFlow.

We next compared SpaGCN, SEDR, SpaceFlow and HyperGCN (BayesSpace does not produce the embeddings) by implementing UMAP visualization [25]. The spots are colored based on the annotation provided in the original publishment of the data. As Fig. 4b shown, HyperGCN can well separate the spots by layers compared to other methods. For other datasets of DLPFC, HyperGCN still have good performance (Additional file 1: Supplementary Figure S1). The results indicate that HyperGCN achieves better visualization embeddings and can be used to implement some downstream analysis.

A domain-specific gene expression analysis was also performed to check the effectiveness of the identified domains from HyperGCN(Fig. 4c). Using the clustering assignments of HyperGCN, the top-1 domain-specific genes for each layer are detected. For example, the domain-specific gene *Saa1* for gray domain (top right, Fig .4a) shows spatial specific expression pattern with high expression level in this domain and low expression outside this domain. For domain-specific gene *Tsmb10*, it has also high expression values in identified domains (layer 5).

### HyperGCN reveals spatial domains of the mouse somatosensory cortex profiled by osmFISH

**Fig. 5:**
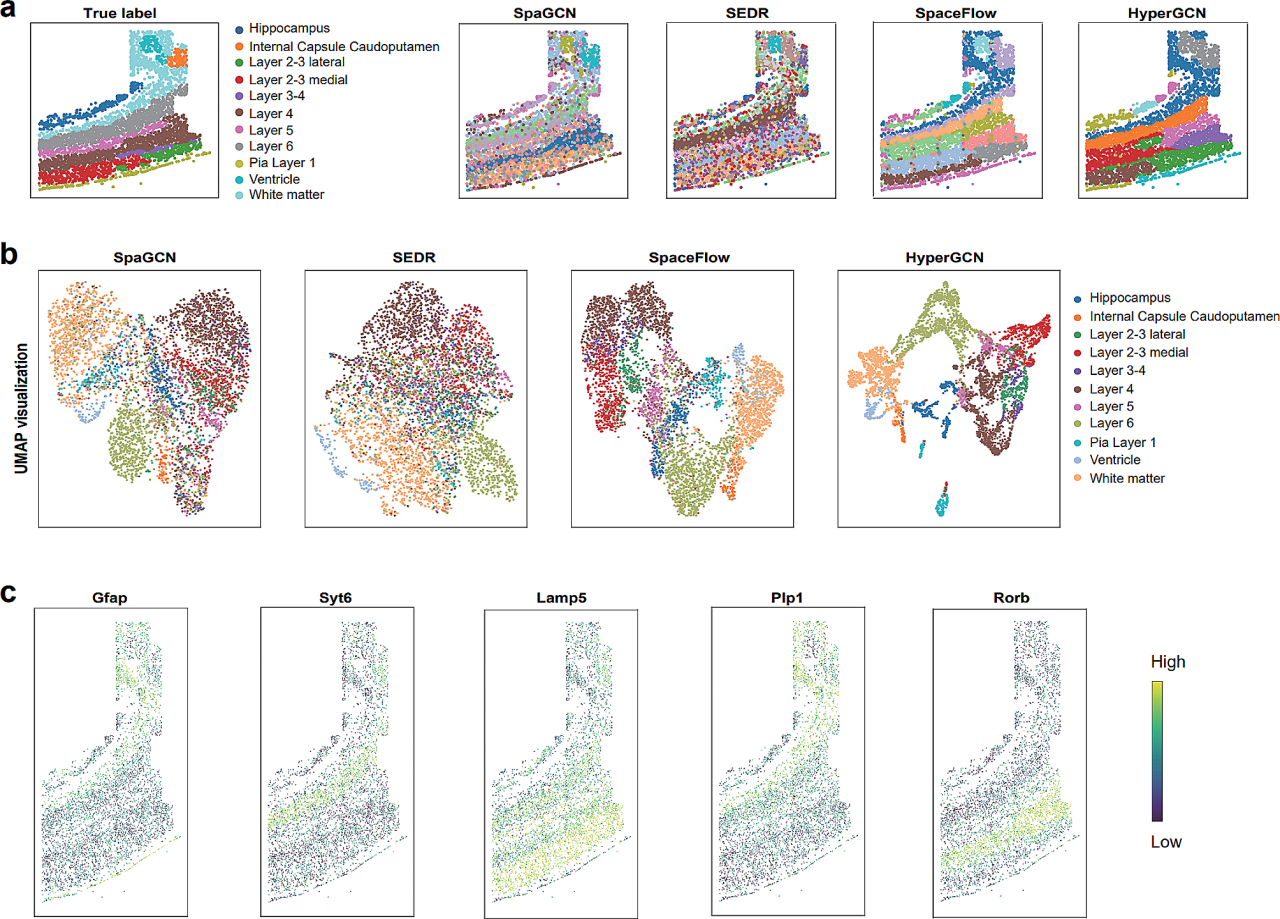
HyperGCN reveals spatial domains of the mouse somatosensory cortex and generates biologically meaningful embeddings on osmFISH data. (**a**) Domain segmentations generated by annotated labels (top left panel) and SpaGCN, SEDR, SpaceFlow and HyperGCN on osmFISH data. (**b**) UMAP visualization using the low-dimensional embeddings from SpaGCN, SEDR, SpaceFlow and HyperGCN. Spots are colored based on their annotated layer labels provided in the original publishment of the data. (**c**) Spatial gene expression heatmap of HyperGCN for the top-1 marker genes of the identified domains

Next, we test whether HyperGCN could provide insights in different tissues profiled by other techniques. We applied HyperGCN onto an osmFISH dataset which contains the gene expressions profiles of the mouse somatosensory cortex section accompanied by spatial information. We found SpaGCN and SEDR identified the roughly domain structures but showed noisy boundaries between layers (Fig. 5a). SpaceFlow captured Pia Layer 1, Layer 6, Layer 2–3 lateral, Ventricle and Internal capsule caudoputamen structures, but also showed an vague and noisy boundaries between domains (Fig. 5a). In contrast, the results of HyperGCN shows a smoother, denoised domain segmentation boundaries and outlines for some layers. Specifically, in hippocampus and Layer 6 region, HyperGCN clearly captured the domain structures which is consistent with the annotation.

We further compare SpaGCN, SEDR, SpaceFlow and HyperGCN by implementing UMAP visualization (Fig. 5b). Spots are colored based on their annotated layer labels provided in the original publishment of the data. We observed that the embeddings of SpaceFlow and HyperGCN could identify the domain spots from different layers. In addition, HyperGCN clearly separated the ventricle and WM domains. This indicates that spatial regularization and hypergraph can encode spatial information and preserve the local and global spatial structure of this data.

We also evaluated the performance of HyperGCN for domain-specific marker gene detection. These structure domains revealed by HyperGCN were clearly supported by the top marker genes of the identified domains (Fig. 5c). We can observed that the top-1 marker genes of the identified domains show spatial specific expression pattern, such as *Gfab*, *Syt6*, *Lamp5*, *Plp1* and *Rorb*. This result is consistent with the original publishment of this data (http://linnarssonlab.org/osmFISH/expression/). This indicates our proposed HyperGCN is effective for the identification of domain structures.

**Fig. 6:**
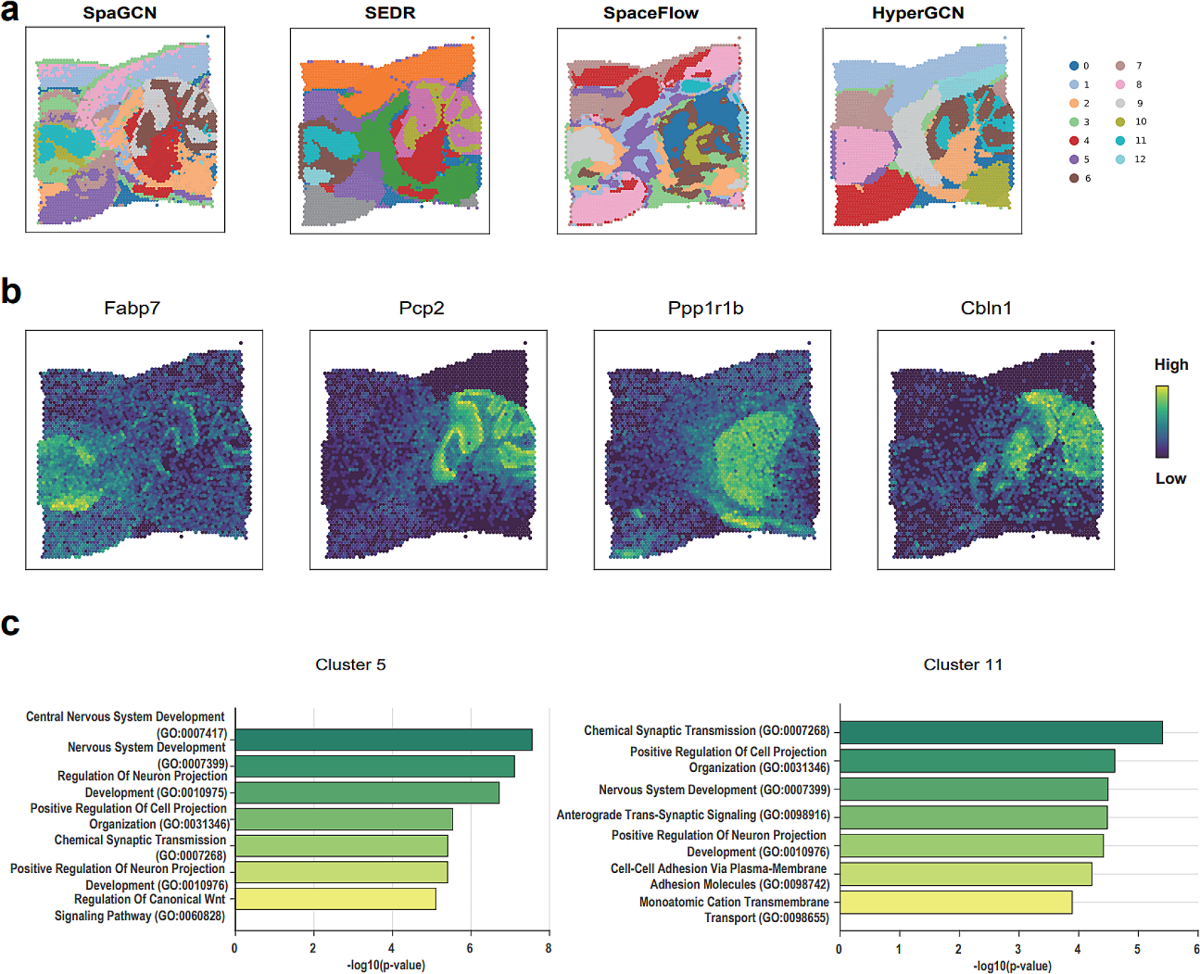
HyperGCN reveals the spatial expression patterns of domain-marker genes on 10X Visium mouse brain sagittal data. (**a**) Domain segmentations generated by SpaGCN, SEDR, SpaceFlow and HyperGCN on 10X Visium mouse brain sagittal data (No annotated labels are provided in the original publishment). (**b**) Spatial gene expression heatmap of HyperGCN for the top marker genes of the identified domains. (**c**) GO enrichment analysis for the domain-specific genes (Top 200) generated from the HyperGCN clustering result with t-test. Enriched terms are showed as -log10(p-value) using Enrichr tool

We also implement experiments on 10X Visium and Stereo-seq datasets which have no ground truth labels provided in this original publishments. The results show HyperGCN achieves consistent good performance in terms of domain segmentation and data visualization (Fig. 6; Additional file 1: Supplementary Figure S2).

### HyperGCN provides rich biological insights on the identified domain structure of the 10X visium mouse brain sagittal data

HyperGCN could uncover spatial gene expressions and provide rich biological insights. We applied HyperGCN in the 10X Visium mouse brain sagittal data to better show the domain structures and spatial expression pattern of genes. We compared the domain segmentation results of SpaGCN, SEDR and SpaceFlow with HyperGCN in the mouse brain sagittal data (Fig. 6a). As expected, HyperGCN exhibited denoised and clean domain structures. Similarly, we also implemented the spatial gene expression analysis of marker genes. More specifically, for the mouse brain sagittal data, we first used Scanpy package [27] to obtain differentially expressed marker genes for each clusters of HyperGCN, and then plotted the spatial gene expression heatmaps using top-1 marker genes of the identified domains. As shown in Fig. 6b, *Pcp2*, *Ppp1r1b*, *Fabp7*, *Cbln1* have high expression scores and show the distinct regional expression patterns in the identified tissue domains. The results are also in accord with SpaGCN and SEDR, which demonstrates that our proposed HyperGCN method can detect the biologically meaningful structure domains on the mouse brain sagittal data profiled with 10X Visium technology.

Other marker genes, the clustering assignment of HyperGCN provided rich information on the biological interpretation of the markers. We performed GO enrichment analysis using Enrichr [43–45]. Top 200 genes with small p-values in each cluster are selected. The enriched terms are in accord with the biological function of the underlying structured domains (Fig. 6c; Additional file 1: Supplementary Table S9). For cluster 7 (pink region in the right subplot of Fig. 6a), GO analysis showed that the marker genes encompassed a wide array of biological processes and pathways such as “Central Nervous System Development” (log10(p-value) = -7.56), “Nervous System Development” (log10(p-value) = -7.11), “Brain Development” (log10(p-value) = -4.28) and “Learning”(log10(p-value) = -4.17). For cluster 10, the enriched BPs “Nervous System Development”(log10(p-value) = -4.50), “Chemical Synaptic Transmission”(log10(p-value) = -5.41), “ Modulation Of Excitatory Postsynaptic Potential”(log10(p-value) = -3.61) are related to synaptic transmission, which may indicate some underlying biological activities.

To summarize, HyperGCN improves the identification of domain structures. The enrichment analyses for the domain-specific marker genes provide consistent and rich biological insights on the detected tissue domains.

### Comparison of computational time

**Fig. 7:**
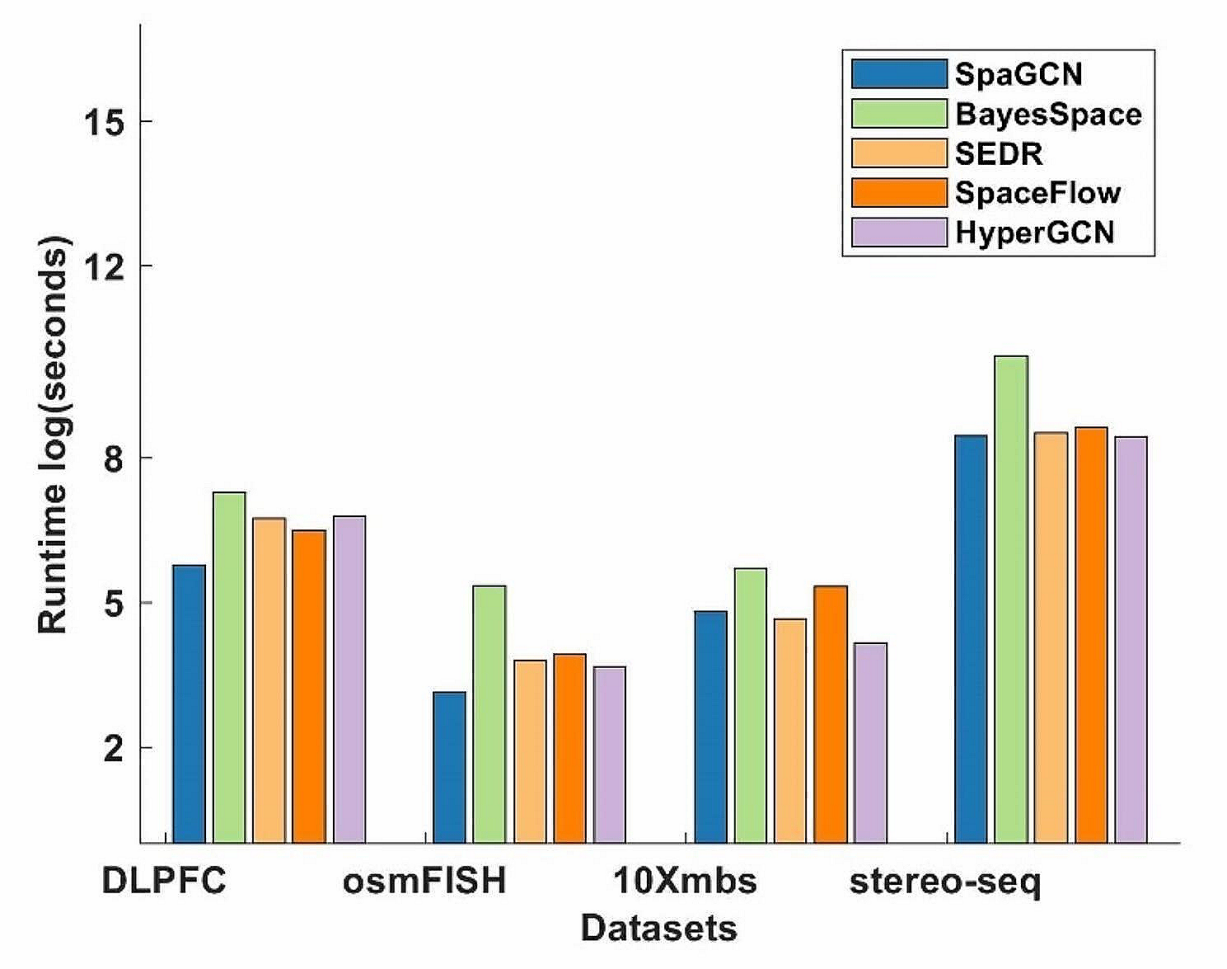
The comparison of runtime of different methods on four real datasets. Experiments on Stereo-seq dataset were run on an i7-11800 H CPU 16 Cores, and 32G RAM. The other experiments in this paper were run on the GeForce RTX 3060 Laptop GPU with 6GB memory

We also compared the computational cost of SpaGCN, BayesSpace, SEDR, SpaceFlow and HyperGCN on four real datasets with different numbers of spots/cells (Fig. 7). For larger datasets (> 20,000 spots), it takes more time to implement BayesSpace. For the DLPFC datasets with 12 spatially resolved RNA-seq data sets, it takes more than 20 min to implement BayesSpace. The computational time for SpaGCN, SEDR, SpaceFlow and HyperGCN is comparable in most datasets. In practice, the run time of HyperGCN on spatial transcriptomics data with 7,000 cells/spots (osmFISH, 10Xmbs) is usually less than 2 min on a GPU. For the whole DLPFC data with 12 spatially resolved RNA-seq datasets, it only takes less than 15 min to implement HyperGCN. For larger datasets (> 10,000 cells/spots), HyperGCN may implement fast on a GPU with large memory.

## Conclusions

Spatially resolved transcriptomics technologies provide an unparalleled opportunity to comprehensively explore transcriptomics data with spatial information. Here we proposed HyperGCN, which integrates the gene expression and spatial proximity information into a hypergraph learning framework. Graph-based methods such as SpaGCN, SEDR and SpaceFlow, utilize nearest neighbors to encode spatial proximity between spots. Unlike these methods, HyperGCN generates a low-dimensional embeddings of spots by hypergraph convolution, which encodes the high-order geometrical structure information of original data into a deep autoencoder clustering framework. Extensive experiments have been conducted on four real spatial transcriptomics datasets. The experimental results show that HyperGCN achieves better clustering performance and clearer domain segmentation, by introducing hypergraph (Fig. 3; Additional file 1: Supplementary Table S2). In addition, HyperGCN facilitates downstream biological analysis, including the characterization of marker genes for the specific domain structure, and GO enrichment analysis. Studying on several real spatial transcriptomics datasets demonstrates the potential of HyperGCN, providing an effective tool to study the difference and functions of domain structures.

The current HyperGCN methodology mainly handles with gene expression and spatial information, and does not consider of histological images [18, 46] and 3D spatial transcriptomics datasets. In the future, we will utilize histological images as an additional modality, and integrate it into the HyperGCN framework to further improve the performance of domain segmentation. An intuitive way is to compute hypergraph by using histology image. Another future direction is to develop new regularization frameworks to adaptively model the spatial distribution patterns and dependencies of different tissues or locations with high geometric complexity [20]. Moreover, integrating single cell RNA data and corresponding spatial information to dissect the mechanism of cell communication is also our future research direction.

### Electronic supplementary material

Below is the link to the electronic supplementary material.

Supplementary Material 1

## Data Availability

The datasets generated or analyzed during the current study are available in the GitHub repository, https://github.com/chonghua-1983/HyperGCN.

## Abbreviations

GCN: Graph Convolutional Network
HyperGCN: Hypergraph induced Graph Convolutional Network
ARI: Adjusted Rand Index
NMI: Normalized Mutual Information
MI: Moran’s Index
UMAP: Uniform Manifold Approximation and Projection
