# Supplementary material for: HyperGCN: an effective deep representation learning framework for the integrative analysis of spatial transcriptomics data
Source: BMC Genomics. 2024 Jun 5;25:566. doi: 10.1186/s12864-024-10469-x (PMC11155133; doi:10.1186/s12864-024-10469-x)
Supplement: Supplementary file 1 — Supplementary Material 1 [file 12864_2024_10469_MOESM1_ESM.docx]

**Additional file 1:** Supplementary materials for “HyperGCN: An Effective Deep Representation Learning Framework for the Integrative Analysis of Spatial Transcriptomics Data”

This file includes the following subsections:

● The comparison between hypergraph and simple graph

● Sensitivity analysis of hyperparameter *k* in hypergraph construction

● Selection of activation functions in HyperGCN

● Robustness analysis of the hyperparameters $\lambda$ and $\gamma$

● Setting of number of centroids in *k*-means step

● The selection of number of clusters

● Supplementary Figures and Tables

● References

**The comparison between hypergraph and simple graph**

In the simple graphs, pairwise interaction graph is used to describe the correlation between any two nodes, and an edge only connects to two nodes. However, a hyperedge can connect to more than two nodes in hypergraph. Intuitively, the hypergraph contains more richer information, direct or potential relationships. Compared to simple graph, the advantages of hypergraph are summarized as follows:

(1) The hypergraph has better interpretability and representation ability. As described in the main text:

*“However, in many real problems, it may cause information loss to represent a group of complex objects only by using simple graph. For example, to group members within one club into different communities, we first construct a simple graph where two members are connected if they share the same coach. Then, clustering methods based on spectral graph are applied. However, this approach mentioned above may lose some useful information in the scenario where the same coach jointly teaches more than two members. Such unexpected information loss may result in the performance degradation of downstream clustering algorithms. Because these members taught by the same coach likely belong to the same community.”*

Hence, A natural way to handle with information loss problem existed in simple graph is to represent the data relationships as hypergraph.

(2) The hypergraph has strict theory deduction as the same as graph Laplacian.

$$O\left( H \right)=\frac{1}{2}\sum_{e\in E} \sum_{\left( i,j \right)\in e} \frac{w\left( e \right)}{\delta\left( e \right)}\left\| H_{i}-H_{j} \right\|_{F}^{2}=Tr\left( H^{T}L_{hyg}H \right).$$

$$L_{hyg}=D_{v}-PWD_{e}^{-1}P^{T}.$$

where $w\left( e \right)$ denotes the weights of hyperedge *e*, $\delta\left( e \right)$ denotes the degree of hyperedge *e*, and $D_{e}$ is the degree matrix of hyperedges. $L_{hyg}$ is hypergraph Laplacian. $H_{i}$ is the low-dimensional representation of node *i*. $d\left( v \right)=\sum_{e\in E} w\left( e \right)p\left( v,e \right)$ is degree matrix of nodes. *P* is the incidence matrix corresponding to hypergraph.

From the equations, we can see that hypergraph Laplacian regularization has the same formalization as the classic graph Laplacian.

(3) We also implement the side-by-side comparison experiments in which classical graph convolutional network (CGCN) and hypergraph convolutional network (HyperGCN) are used to enforce the clustering structure. The experimental results show that HyperGCN has better performance on ARI, NMI, and Moran Index metrics.

|  | DLPFC | | | osmFISH | | | 10Xmbs | Stereo-seq |
| --- | --- | --- | --- | --- | --- | --- | --- | --- |
|  | ARI | NMI | Moran | ARI | NMI | Moran | Moran | Moran |
| CGCN | 0.3994 | 0.5590 | **0.8939** | 0.5257 | 0.6445 | 0.9001 | 0.5866 | 0.8451 |
| HyperGCN | **0.4395** | **0.5874** | 0.8913 | **0.5625** | **0.6611** | **0.9003** | **0.7537** | **0.8853** |

**Sensitivity analysis of hyperparameter *k* in hypergraph construction**

In the manuscript, we use the spatial coordinates of spots to construct the *k*NN graph, and then a hypergraph is constructed based on the *k*NN graph with the methodology proposed in the main text. In the whole experiments, we set $k=20$ for all the datasets. To test the robustness of HyperGCN on the hyperparameter $k$, we varied $k$ in the range $\left\{ 10,20, 30,40,50 \right\}$. The results are as follows.

| *k* | DLPFC | | | osmFISH | | | 10Xmbs | Stereo-seq |
| --- | --- | --- | --- | --- | --- | --- | --- | --- |
|  | ARI | NMI | Moran | ARI | NMI | Moran | Moran | Moran |
| 10 | 0.4304 | 0.5822 | 0.9123 | 0.5813 | 0.6942 | 0.8405 | 0.7035 | 0.8438 |
| 20 | 0.4395 | 0.5874 | 0.8913 | 0.5625 | 0.6611 | 0.9003 | 0.7537 | 0.8853 |
| 30 | 0.3859 | 0.5397 | 0.8714 | 0.6142 | 0.7458 | 0.8492 | 0.7215 | 0.8880 |
| 40 | 0.3944 | 0.5555 | 0.8958 | 0.5144 | 0.7013 | 0.8685 | 0.7306 | 0.8686 |
| 50 | 0.4135 | 0.5745 | 0.8909 | 0.5709 | 0.6999 | 0.8894 | 0.7401 | 0.8779 |

As the table shown, the different values of *k* in hypergraph construction led to the change of performance of the model. Especially, when the number of cells is small (<5000), the performance of HyperGCN seems less stable. However, when the scale of dataset is large, the performance of HyperGCN is relatively stable with k varies (Stereo-seq data, >19527). In the manuscript, for the sake of fairness, we choose the same $k$ in all the real datasets that we have implemented.

**Selection of activation functions in HyperGCN**

In the manuscript, we used Elu activation function in the autoencoder. Overall, Elu has some advantages as follows:

(1) Compared with other activation functions, Elu does not suffer from the problem of vanishing gradients and exploding gradients. Unlike ReLU, Elu does not suffer from the problems of dying neurons. It has proven to better than ReLU and Leaky-Relu and Parameterized-ReLU. Using Elu leads to a lower training times and higher accuracies compared to ReLU and its variants.

The Elu activation function can be written as:

$$Elu\left( x \right)=\left\{ \begin{aligned} \exp\left( x \right)-1; if x<0 \\ x; if x\geq0 \end{aligned} \right.$$

(2) Unlike ReLU, Elu has a negative value too which cause the mean of the Elu activation function to shift towards 0. Clevert et.al argued that due to this shift, the training of the model converges faster than other activations. Moreover, Elu has also better generalized performance.

**Robustness analysis of the hyperparameters** $\boldsymbol{\lambda}$ **and** $\boldsymbol{\gamma}$

For $\lambda$ and γ, we set the values of $\lambda$ and γ are the same in all the real datasets that we have implemented. To test the robustness of HyperGCN on the hyperparameters, we varied $\lambda$ and γ in the range $\left\{ \lambda^{*}/10,\lambda^{*}/5, \lambda^{*}/2,\lambda^{*}, 2\lambda^{*},5\lambda^{*}, 10\lambda^{*}, \right\}$ and$\left\{ \gamma^{*}/10,\gamma^{*}/5,\gamma^{*}/2,\gamma^{*}, 2\gamma^{*},5\gamma^{*}, 10\gamma^{*} \right\}$, respectively. Here $\lambda^{*}$ and $\gamma^{*}$ are the hyperparameters chosen in our manuscript.

$$L={{Loss}_{rec}+\lambda Loss}_{clu}+{\gamma Loss}_{spa}$$

The results are presented in Supplementary Table S7-S8. We can see that the performance of HyperGCN is stable in most cases when $\lambda$ and γ vary. In the revised manuscript, these results have been presented in section “Conclusions”.

**Setting of number of centroids in *k*-means step**

In the manuscript, *k*-means is used to enhance the initialization step of clustering. The number of centroids in k-means is empirically set as 10 for all datasets. We tested the robustness of HyperGCN on the number of centroids in *k*-means, where we varied the number of centroids from 5 to 50. The clustering performance assessed by NMI, ARI and Moran Index are as follows.

| No. of centroids | DLPFC | | | osmFISH | | | 10Xmbs | Stereo-seq |
| --- | --- | --- | --- | --- | --- | --- | --- | --- |
|  | ARI | NMI | Moran | ARI | NMI | Moran | Moran | Moran |
| 5 | 0.4331 | 0.5814 | 0.8858 | 0.5654 | 0.6749 | 0.8840 | 0.7258 | 0.8489 |
| 10 | 0.4395 | 0.5874 | 0.8913 | 0.5625 | 0.6611 | 0.9003 | 0.7537 | 0.8853 |
| 20 | 0.4124 | 0.5689 | 0.8819 | 0.5150 | 0.6796 | 0.9000 | 0.7453 | 0.8678 |
| 30 | 0.4373 | 0.5769 | 0.8865 | 0.4231 | 0.5965 | 0.8681 | 0.7345 | 0.8751 |
| 50 | 0.4481 | 0.5850 | 0.8869 | 0.4942 | 0.6376 | 0.8991 | 0.7082 | 0.8825 |

As the table shown, in most cases, the performance is robust to the number of centroids. For the osmFISH dataset, ARI and NMI metric seems to less robust, which is likely because spatial transcriptomics data tends to have high level of noise. We suggest that setting the default number of centroids in *k*-means to be 10 for a dataset with unknown number of factors.

**The selection of number of clusters**

In the manuscript, we set the number of clusters equal to the numbers of annotated layers for DLFPC data and osmFISH data. The numbers of factors were chosen as the following:

DLPFC:

5: '151669', '151670', '151671', '151672'

7: '151507', '151508', '151509', '151510', '151673', '151674', '151675', '151676'

osmFISH: 11

We tested the robustness of HyperGCN on the number of clusters, where we varied the number of clusters in the range {5,6,7,8,9,10} and {8,9,10,11,12} on DLPFC and osmFISH data, respectively. The clustering performance evaluated by NMI, ARI and Moran are presented in Supplementary Table S9. For DLPFC data, the NMI and ARI is robust to the number of clusters. For osmFISH data, when the number of clusters equal to be 10, the performance is the best.

We also tested several existing methods to determine the number of clusters in the non-negative matrix factorization. Specifically, we first compute the Laplacian matrix of gene expression. Based on the eigenvalue decomposition of the Laplacian matrix, a sorted eigenvalues list can be obtained. Then, we compute the gap between two consecutive eigenvalues and select the biggest gap as the number of clusters. However, we found that these methods tend to give a smaller number of clusters than the true number of cell types, which is likely because the spatial transcriptomics data tends to have high level of noise. For a dataset with unknown number of sample types, we suggest that using unsupervised metric Moran Index to select the number of clusters, i.e., by varying the range of cluster number, selecting the one with the highest Moran values.

**Supplementary Figures and Tables**


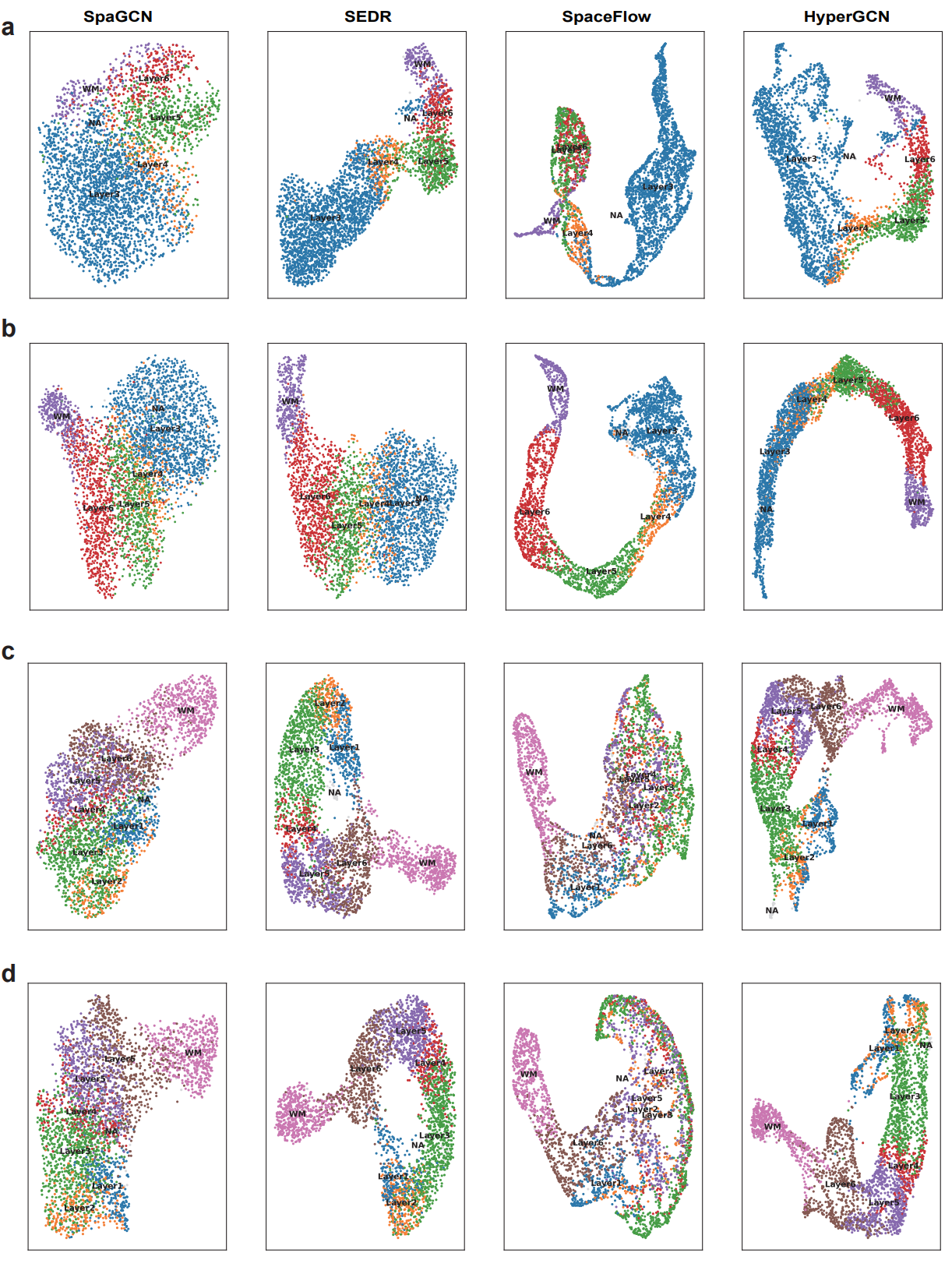


**Supplementary Figure S1:** UMAP visualization on DLPFC data. a) 151670. b) 151672. c) 151674. d) 151676.

**
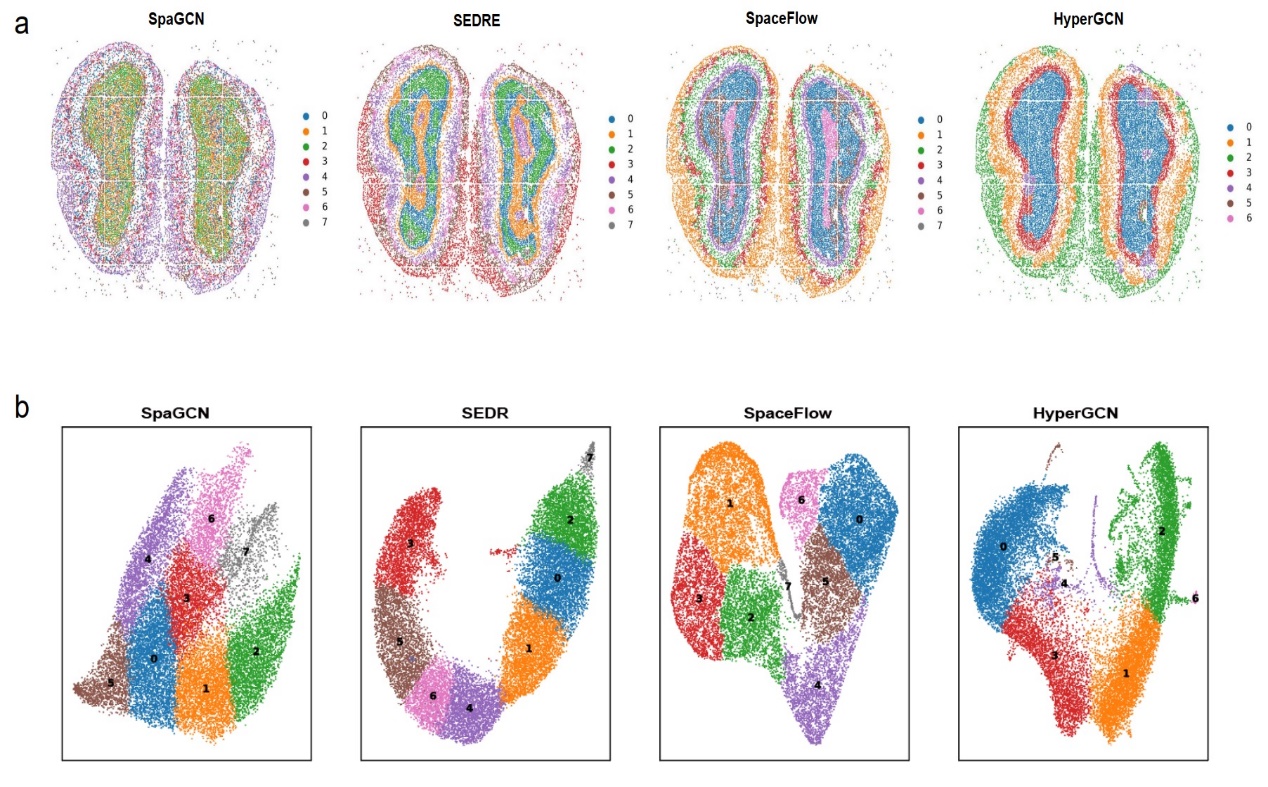
**

**Supplementary Figure S2:** Analysis on Stereo-seq data. a) Domain segmentation generated by SpaGCN, SEDR, SpaceFlow and HyperGCN on Stereo-seq data (no annotated labels are provided in the original data). b) UMAP visualization using the low-dimensional embeddings from SpaGCN, SEDR, SpaceFlow and HyperGCN. Spots are colored based on their predicted layer labels.

**Supplementary Table S1:** Statistical information of datasets

| Datasets | | # Cells/Spots | # Features |
| --- | --- | --- | --- |
| DLPFC | 151507 | 4226 | 33538 |
|  | 151508 | 4384 | 33538 |
|  | 151509 | 4789 | 33538 |
|  | 151510 | 4634 | 33538 |
|  | 151669 | 3661 | 33538 |
|  | 151670 | 3498 | 33538 |
|  | 151671 | 4110 | 33538 |
|  | 151672 | 4015 | 33538 |
|  | 151673 | 3639 | 33538 |
|  | 151674 | 3673 | 33538 |
|  | 151675 | 3592 | 33538 |
|  | 151676 | 3460 | 33538 |
| osmFISH | | 4839 | 33 |
| 10Xmbs | | 6112 | 32285 |
| Stereo-seq | | 19527 | 22795 |

**Supplementary Table S2:** Clustering performance on four real datasets

|  | DLPFC | | | osmFISH | | | 10Xmbs | Stereo-seq |
| --- | --- | --- | --- | --- | --- | --- | --- | --- |
|  | ARI | NMI | Moran | ARI | NMI | Moran | Moran | Moran |
| SpaGCN | 0.3884 | 0.5287 | 0.7665 | 0.2606 | 0.3286 | 0.4182 | 0.3890 | 0.3303 |
| BayesSpace | 0.4466 | 0.6103 | 0.8720 | 0.3546 | 0.3982 | 0.4519 | 0.5386 | 0.3429 |
| SEDR | 0.4158 | 0.5392 | 0.7373 | 0.2391 | 0.3101 | 0.3631 | 0.5659 | 0.7828 |
| SpaceFlow | 0.2782 | 0.4450 | 0.7291 | 0.4869 | 0.5945 | 0.7738 | 0.6537 | 0.6716 |
| HyperGCN | 0.4395 | 0.5874 | 0.8913 | 0.5625 | 0.6611 | 0.9003 | 0.7537 | 0.8853 |

**Supplementary Table S3:** Clustering performance on other real datasets from different sequencing techniques and tissues.

|  | seqFISH+  Mouse  Brain cortex | Human  (positive Breast Tumors) | 10X Xenium  Human  (Colon Cancer) | 10X Visium  Zabrafish  (melanoma) |
| --- | --- | --- | --- | --- |
|  | Moran | Moran | Moran | Moran |
| SpaGCN | 0.1710 | 0.5814 | 0.5693 | 0.6398 |
| BayesSpace | 0.1558 | 0.6270 | 0.7913 | 0.8117 |
| SEDR | 0.3954 | 0.5214 | 0.7312 | 0.7675 |
| SpaceFlow | 0.2398 | 0.7609 | 0.8049 | 0.7368 |
| HyperGCN | 0.6290 | 0.7354 | 0.8216 | 0.8747 |

**Supplementary Table S4:** Assessment of clustering performance when benchmarked with other state-of-the-art methods

|  | DLPFC | | | osmFISH | | | 10Xmbs | Stereo-seq |
| --- | --- | --- | --- | --- | --- | --- | --- | --- |
|  | ARI | NMI | Moran | ARI | NMI | Moran | Moran | Moran |
| SOTIP | 0.2147 | 0.3399 | 0.8067 | 0.5623 | 0.6403 | 0.7609 | 0.7371 | 0.6901 |
| STAGATE | 0.4872 | 0.6466 | 0.8583 | 0.1605 | 0.1231 | 0.3527 | 0.7735 | 0.6608 |
| GraphST | 0.4109 | 0.5598 | 0.7499 | 0.4384 | 0.5432 | 0.6998 | 0.6109 | 0.4843 |
| DR_SC | 0.4124 | 0.5672 | 0.8453 | 0.1488 | 0.2180 | 0.2518 | 0.5325 | 0.2211 |
| HyperGCN | 0.4395 | 0.5874 | 0.8913 | 0.5625 | 0.6611 | 0.9003 | 0.7537 | 0.8853 |

**Supplementary Table S5:** The performance of HyperGCN when $\gamma$ varies. $\gamma^{*}$ is chosen in HyperGCN for all the datasets.

| $\gamma$ | DLPFC | | | osmFISH | | | 10Xmbs | Stereo-seq |
| --- | --- | --- | --- | --- | --- | --- | --- | --- |
|  | ARI | NMI | Moran | ARI | NMI | Moran | Moran | Moran |
| $\gamma^{*}/10$ | 0.4296 | 0.5809 | 0.8911 | 0.5410 | 0.6630 | 0.8803 | 0.7053 | 0.8608 |
| $\gamma^{*}/5$ | 0.4015 | 0.5735 | 0.8888 | 0.6135 | 0.6787 | 0.8688 | 0.7152 | 0.8764 |
| $\gamma^{*}/2$ | 0.4195 | 0.5673 | 0.8918 | 0.5553 | 0.6525 | 0.8686 | 0.7285 | 0.8806 |
| $\gamma^{*}$ | 0.4395 | 0.5874 | 0.8913 | 0.5625 | 0.6611 | 0.9003 | 0.7537 | 0.8853 |
| $2\gamma^{*}$ | 0.4506 | 0.5884 | 0.8980 | 0.5300 | 0.6822 | 0.8718 | 0.7496 | 0.8810 |
| $5\gamma^{*}$ | 0.3949 | 0.5446 | 0.8619 | 0.5308 | 0.6713 | 0.9097 | 0.7365 | 0.8728 |
| $10\gamma^{*}$ | 0.4295 | 0.5807 | 0.8925 | 0.5212 | 0.6615 | 0.9001 | 0.7189 | 0.8486 |

**Supplementary Table S6:** The performance of HyperGCN when $\lambda$ varies. $\lambda^{*}$ is chosen in HyperGCN for all the datasets.

| $\lambda$ | DLPFC | | | osmFISH | | | 10Xmbs | Stereo-seq |
| --- | --- | --- | --- | --- | --- | --- | --- | --- |
|  | ARI | NMI | Moran | ARI | NMI | Moran | Moran | Moran |
| $\lambda^{*}/10$ | 0.4242 | 0.5776 | 0.8859 | 0.5593 | 0.6876 | 0.8802 | 0.6963 | 0.8614 |
| $\lambda^{*}/5$ | 0.4246 | 0.5787 | 0.8951 | 0.5829 | 0.6771 | 0.8607 | 0.7230 | 0.8772 |
| $\lambda^{*}/2$ | 0.4284 | 0.5748 | 0.9099 | 0.5328 | 0.6610 | 0.8885 | 0.7352 | 0.8791 |
| $\lambda^{*}$ | 0.4395 | 0.5874 | 0.8913 | 0.5625 | 0.6611 | 0.9003 | 0.7537 | 0.8853 |
| $2\lambda^{*}$ | 0.4418 | 0.5927 | 0.8863 | 0.5520 | 0.6274 | 0.8721 | 0.7216 | 0.8623 |
| $5\lambda^{*}$ | 0.4080 | 0.5727 | 0.8929 | 0.5860 | 0.6923 | 0.8763 | 0.7069 | 0.8302 |
| $10\lambda^{*}$ | 0.3995 | 0.5700 | 0.8900 | 0.5430 | 0.6738 | 0.8760 | 0.6879 | 0.8536 |

**Supplementary Table S7:** The performance test of HyperGCN for different clusters

| DLPFC | | | | osmFISH | | | |
| --- | --- | --- | --- | --- | --- | --- | --- |
| Clusters (#) | ARI | NMI | Moran | Clusters (#) | ARI | NMI | Moran |
| 6 | 0.4105 | 0.5737 | 0.8890 | 8 | 0.5720 | 0.6591 | 0.8876 |
| Number of annotated layers:  5, 7 for different dataset | 0.4395 | 0.5874 | 0.8913 | 9 | 0.5604 | 0.6834 | 0.8924 |
| 8 | 0.4031 | 0.5809 | 0.8763 | 10 | 0.6035 | 0.6896 | 0.9048 |
| 9 | 0.3653 | 0.5649 | 0.8525 | Number of annotated layers: 11 | 0.5625 | 0.6611 | 0.9003 |
| 10 | 0.3494 | 0.5528 | 0.8275 | 12 | 0.5821 | 0.6847 | 0.8412 |

**Supplementary Table S8:** The performance of HyperGCN by implementing ablation experiments

|  | DLPFC | | | osmFISH | | | 10Xmbs | Stereo-seq |
| --- | --- | --- | --- | --- | --- | --- | --- | --- |
|  | ARI | NMI | Moran | ARI | NMI | Moran | Moran | Moran |
| $\lambda=0$ | 0.4355 | 0.5759 | 0.8924 | 0.5212 | 0.6686 | 0.9070 | 0.8087 | 0.8633 |
| $\gamma=0$ | 0.4328 | 0.5784 | 0.8856 | 0.4692 | 0.6179 | 0.8390 | 0.6643 | 0.8420 |
| HyperGCN | 0.4395 | 0.5874 | 0.8913 | 0.5625 | 0.6611 | 0.9003 | 0.7537 | 0.8853 |

**Supplementary Table S9:** Enriched significant GO biological processes/pathways in the Enrichr(Chen, et al., 2013; Kuleshov, et al., 2016; Xie, et al., 2021) analysis for the differentially expressed marker genes of the identified domain on the 10X Visium mouse brain sagittal data. Log10(p-value) is the p-value in log base 10. The BPs/pathways are sorted by p-value and only the top pathways with log10(p-value) < -4 are shown.

| **Cluster** | **GO biological process/pathway** | **Log(p-value)** | **Log**  **(adjusted p-value)** |
| --- | --- | --- | --- |
| Cluster 7 | Nervous System Development (GO:0007399) | -6.41 | -3.33 |
|  | 'Central Nervous System Development (GO:0007417) | -5.24 | -2.49 |
|  | 'Myelination (GO:0042552) | -5.10 | -2.49 |
|  | 'Adenylate Cyclase-Inhibiting G Protein-Coupled Receptor Signaling Pathway (GO:0007193) | -4.89 | -2.41 |
|  | Axon Ensheathment In Central Nervous System (GO:0032291) | -4.48 | -2.09 |
| Cluster 10 | Modulation Of Chemical Synaptic Transmission (GO:0050804) | -7.36 | -4.24 |
|  | Chemical Synaptic Transmission (GO:0007268) | -6.95 | -4.14 |
|  | Negative Regulation Of Cation Channel Activity (GO:2001258) | -6.54 | -3.90 |
|  | Regulation Of Potassium Ion Transport (GO:0043266) | -6.35 | -3.83 |
|  | Anterograde Trans-Synaptic Signaling (GO:0098916) | -6.095 | -3.68 |
|  | Regulation Of Synaptic Transmission, Glutamatergic (GO:0051966) | -5.915 | -3.58 |
|  | Regulation Of Long-Term Neuronal Synaptic Plasticity (GO:0048169) | -5.715 | -3.44 |
|  | 'Negative Regulation Of Calcium Ion Transmembrane Transporter Activity (GO:1901020)' | -4.96 | -2.74 |
|  | Adenylate Cyclase-Inhibiting G Protein-Coupled Receptor Signaling Pathway (GO:0007193) | -4.89 | -2.73 |
|  | Regulation Of Cation Channel Activity (GO:2001257) | -4.70 | -2.61 |
|  | Regulation Of Neuronal Synaptic Plasticity (GO:0048168) | -4.68 | -2.61 |
|  | Glutamate Receptor Signaling Pathway (GO:0007215) | -4.62 | -2.60 |
|  | Positive Regulation Of GTPase Activity (GO:0043547) | -4.60 | -2.60 |
|  | Negative Regulation Of Calcium Ion Transmembrane Transport (GO:1903170) | -4.48 | -2.51 |
|  | Positive Regulation Of Cation Channel Activity (GO:2001259) | -4.44 | -2.50 |
|  | Regulation Of Monoatomic Cation Transmembrane Transport (GO:1904062) | -4.38 | -2.47 |
|  | Regulation Of GTPase Activity (GO:0043087) | -4.21 | -2.33 |
|  | Regulation Of Neuron Projection Development (GO:0010975) | -4.18 | -2.33 |
|  | Regulation Of G Protein-Coupled Receptor Signaling Pathway (GO:0008277) | -4.15 | -2.33 |
|  | Positive Regulation Of Hydrolase Activity (GO:0051345) | -4.14 | -2.33 |
|  | Response To Calcium Ion (GO:0051592) | -4.02 | -2.23 |

**References**

Chen, E.Y.*, et al.* Enrichr: interactive and collaborative HTML5 gene list enrichment analysis tool. *BMC bioinformatics* 2013;14(1):1-14.

Kuleshov, M.V.*, et al.* Enrichr: a comprehensive gene set enrichment analysis web server 2016 update. *Nucleic acids research* 2016;44(W1):W90-W97.

Xie, Z.*, et al.* Gene set knowledge discovery with Enrichr. *Current protocols* 2021;1(3):e90.

Yuan Z, Li Y, Shi M, et al. SOTIP is a versatile method for microenvironment modeling with spatial omics data[J]. Nature Communications, 2022, 13(1): 7330.

Dong K, Zhang S. Deciphering spatial domains from spatially resolved transcriptomics with an adaptive graph attention auto-encoder[J]. Nature communications, 2022, 13(1): 1739.

Long Y, Ang K S, Li M, et al. Spatially informed clustering, integration, and deconvolution of spatial transcriptomics with GraphST[J]. Nature Communications, 2023, 14(1): 1155.

Liu W, Liao X, Yang Y, et al. Joint dimension reduction and clustering analysis of single-cell RNA-seq and spatial transcriptomics data[J]. Nucleic acids research, 2022, 50(12): e72-e72.

Clevert D A, Unterthiner T, Hochreiter S. Fast and accurate deep network learning by exponential linear units (elus)[J]. arXiv preprint arXiv:1511.07289, 2015.

Von Luxburg U. A tutorial on spectral clustering[J]. Statistics and computing, 2007, 17(4): 395-416.

Meyer C, Race S, Valakuzhy K. Determining the number of clusters via iterative consensus clustering[C] Proceedings of the 2013 SIAM International Conference on Data Mining. Society for Industrial and Applied Mathematics, 2013: 94-102.
